# 2-Chloro-*N*-[4-(3-methyl-3-phenyl­cyclo­but­yl)-1,3-thia­zol-2-yl]-*N*′-(naphthalen-1-yl­methyl­idene)acetohydrazide

**DOI:** 10.1107/S1600536811000183

**Published:** 2011-01-08

**Authors:** Ersin Inkaya, Muharrem Dinçer, Alaaddin Çukurovalı, Engin Yılmaz

**Affiliations:** a Department of Physics, Arts and Sciences Faculty, Ondokuz Mayıs University, 55139 Samsun, Turkey; b Department of Chemistry, Science Faculty, Fırat University, 23119 Elazığ, Turkey; c Department of Chemistry, Science Faculty, Bitlis Eren University, 13000 Bitlis, Turkey

## Abstract

In the mol­ecular structure of the title hydrazide derivative, C_27_H_24_ClN_3_OS, the acetohydrazide group is approximately planar, with a maximum deviation of 0.017 (3) Å. The dihedral angle between the naphthyl­ene system and the phenyl ring is 78.91 (18)°. The crystal structure is stabilized by one weak inter­molecular C—H⋯O hydrogen bond and two aliphatic C—H⋯π hydrogen-bonding associations.

## Related literature

For the applications and bioactivity of hydrazide derivatives, see: Feng *et al.* (2006 ▶); Yang *et al.* (2007 ▶); Kamal *et al.* (2007 ▶); Masunari & Tavares (2007 ▶); Rando *et al.* (2002 ▶). For bond-length data, see: Demir *et al.* (2006 ▶).
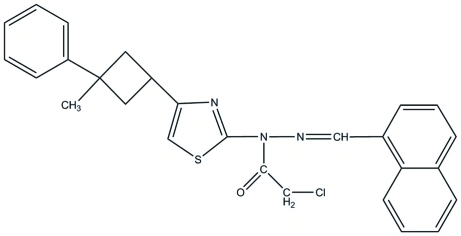


## Experimental

### 

#### Crystal data

C_27_H_24_ClN_3_OS
*M* *_r_* = 474.00
Monoclinic, 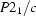

*a* = 7.498 (5) Å
*b* = 12.823 (5) Å
*c* = 24.924 (5) Å
β = 92.185 (5)°
*V* = 2394.6 (19) Å^3^
*Z* = 4
Mo *K*α radiation
μ = 0.27 mm^−1^
*T* = 296 K
0.36 × 0.22 × 0.12 mm

#### Data collection

Stoe IPDS 2 CCD diffractometer
Absorption correction: integration (*X-RED32*; Stoe & Cie, 2002 ▶) *T*_min_ = 0.797, *T*_max_ = 0.961
12524 measured reflections
4206 independent reflections
1375 reflections with *I* > 2σ(*I*)
*R*_int_ = 0.135

#### Refinement

*R*[*F* ^2^ > 2σ(*F* ^2^)] = 0.069
*wR*(*F* ^2^) = 0.156
*S* = 0.90
4206 reflections
298 parameters
H-atom parameters constrained
Δρ_max_ = 0.14 e Å^−3^
Δρ_min_ = −0.16 e Å^−3^

### 

Data collection: *X-AREA* (Stoe & Cie, 2002 ▶); cell refinement: *X-AREA*; data reduction: *X-RED32* (Stoe & Cie, 2002 ▶); program(s) used to solve structure: *SHELXS97* (Sheldrick, 2008 ▶); program(s) used to refine structure: *SHELXL97* (Sheldrick, 2008 ▶); molecular graphics: *ORTEP-3 for Windows* (Farrugia, 1997 ▶); software used to prepare material for publication: *WinGX* (Farrugia, 1999 ▶) and *PLATON* (Spek, 2009 ▶).

## Supplementary Material

Crystal structure: contains datablocks global, I. DOI: 10.1107/S1600536811000183/zs2087sup1.cif

Structure factors: contains datablocks I. DOI: 10.1107/S1600536811000183/zs2087Isup2.hkl

Additional supplementary materials:  crystallographic information; 3D view; checkCIF report

## Figures and Tables

**Table 1:** Hydrogen-bond geometry (Å, °) *Cg*1 and *Cg*2 are the centroids of the C12/C13/S1/C14/N1 and C22–C27 rings, respectively.

*D*—H⋯*A*	*D*—H	H⋯*A*	*D*⋯*A*	*D*—H⋯*A*
C13—H13⋯O1^i^	0.93	2.41	3.243 (7)	148
C8—H7*A*⋯*Cg*1^ii^	0.96	2.94	3.748 (3)	143
C16—H16*B*⋯*Cg*2^iii^	0.97	2.93	3.740 (8)	142

Symmetry codes: (i) 
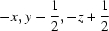
; (ii) 
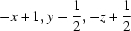
; (iii) 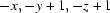.

**Fig. 1.**
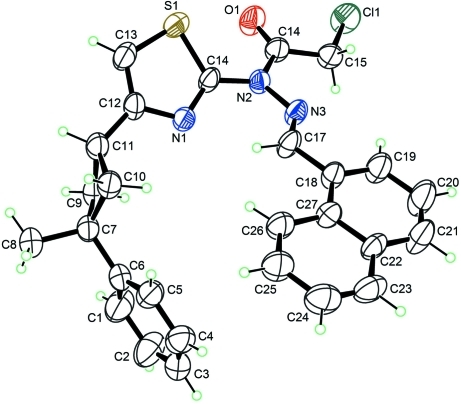
An ORTEP-3 (Farrugia, 1997) drawing of (I), showing the atomic numbering scheme. Displacement ellipsoids are drawn at the 30% probability level.

**Fig. 2.**
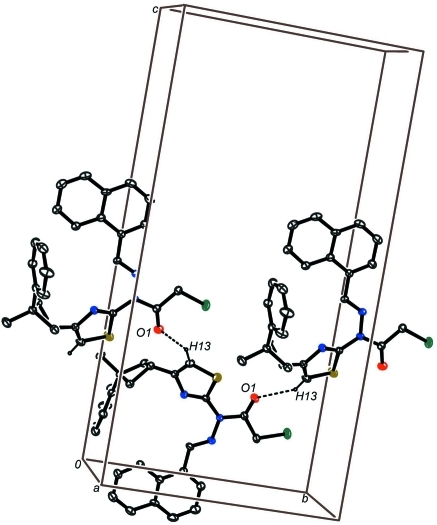
Part of the crystal structure of the title compound, showing the C—H···O hydrogen bonding. For clarity, only H atoms involved in hydrogen bonding have been included. For symmetry codes, see Table 1.

**Fig. 3.**
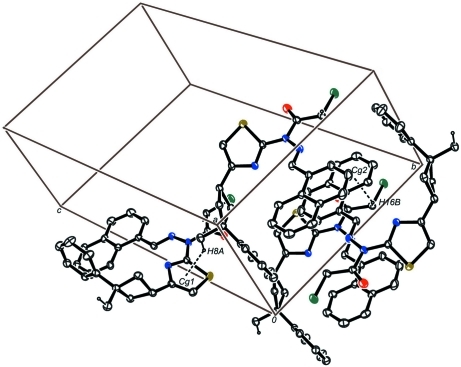
Part of the crystal structure of the title compound, showing the C—H···π interactions. For clarity, only H atoms involved in hydrogen bonding have been included. For symmetry codes, see Table 1.

**Fig. 4.**
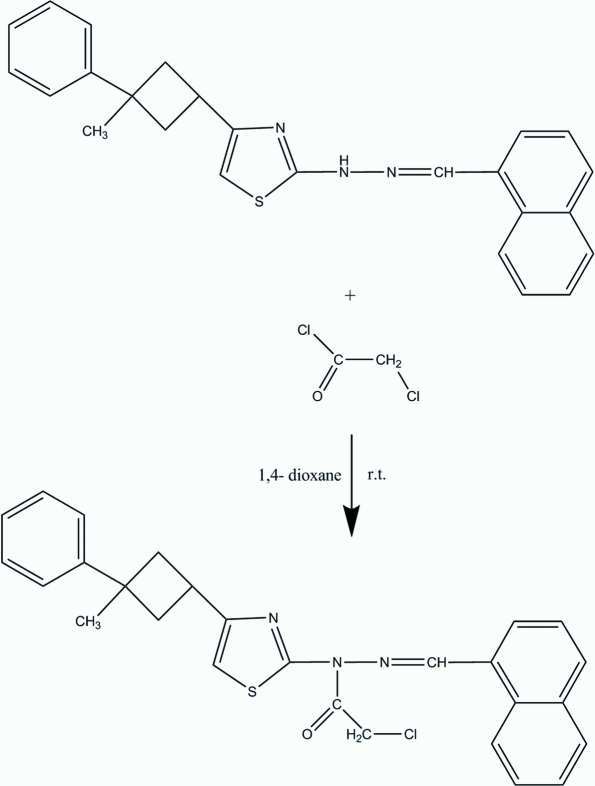
Reaction scheme for the title compound.

## supplementary crystallographic information

### Comment

Hydrazide derivatives of various compounds are very important units in host–guest chemistry due to their special arrangement of donor-acceptor functional groups (Feng *et al.*, 2006; Yang *et al.*, 2007). Hydrazine derivatives have also been associated with remarkable anticancer (Kamal *et al.*, 2007), antibacterial (Masunari & Tavares, 2007) and tuberculostatic (Rando *et al.*, 2002) activities. The title compound, the hydrazide derivative C_27_H_24_N_3_OClS (I) has been synthesized and its crystal structure is reported here.

In the structure of (I) (Fig. 1) the phenyl and thiazole rings are *cis*-related with respect to the cyclobutane ring. The dihedral angle between the naphthylene fragment with the thiazole and phenyl rings are 35.76 (17)° and 78.91 (18)°, respectively. The cyclobutane ring is puckered, with a dihedral angle of 25.20 (5)° between the two three-membered halves of the ring. The dihedral angle between the acetohydrazide group and the thiazole ring is 32.28 (38)°. The C═O bond distance is 1.190 (6) Å comparing with a literature value of 1.187 (16) Å (Demir *et al.*, 2006).

The crystal packing involves a weak intermolecular thiazole C13—H···O1 hydrogen bond (Table 1, Fig. 2) and intermoleculer C8—H···π (thiazole ring C12/C13/S1/C14/N1), C16—H···π (ring C22—C27) hydrogen-bonding associations (Fig. 3).

### Experimental

The synthesis of the title compound was simply carried out in the following reaction (Fig. 4). A solution of 0.3975 gram (1 mmol) of *N*-[4-(3-methyl-3-phenyl-cyclobutyl)-thiazol-2-yl]-*N*-naphthalen -1-ylmethylenehydrazine was dissolved in 20 ml of dioxane containing 1 mmol triethylamine. To this solution, 90 µ*L* (1 mmol) of chloroacetyl chloride solution in 20 ml 1,4-dioxane was added dropwise over a two hour period at room temperature with stirring. Mixture was stirred two hours more and then neutralized with 5% aqueous ammonia. The compound thus precipitated was filtered, washed with copious water and crystallized from ethanol, giving brown crystals (yield, 93%).

### Refinement

The data was poor because of the weakly diffracting crystals which were not of good quality. Although a long exposure time (5 minute) was applied, the reflections were quite weak, resulting in a too low observed/unique reflection ratio. H atoms were positioned geometrically and treated using a riding model, fixing the bond lengths at 0.96, 0.97, 0.98 and 0.93 Å for CH_3_, CH_2_, CH and CH(aromatic), respectively. The displacement parameters of the H atoms were constrained with *U*_iso_(H) = 1.2*U*_eq_ (aromatic, methylene or methine C) or 1.5*U*_eq_ (methyl C).

### Crystal data

**Table:**

C_27_H_24_ClN_3_OS	*F*(000) = 992
*M**_r_* = 474.00	*D*_x_ = 1.315 Mg m^−^^3^
Monoclinic, *P*2_1_/*c*	Mo *K*α radiation, λ = 0.71069 Å
Hall symbol: -P 2ybc	Cell parameters from 9611 reflections
*a* = 7.498 (5) Å	θ = 1.6–27.3°
*b* = 12.823 (5) Å	µ = 0.27 mm^−^^1^
*c* = 24.924 (5) Å	*T* = 296 K
β = 92.185 (5)°	Prism, pale brown
*V* = 2394.6 (19) Å^3^	0.36 × 0.22 × 0.12 mm
*Z* = 4	

### Data collection

**Table:**

Stoe IPDS 2 CCD diffractometer	4206 independent reflections
Radiation source: fine-focus sealed tube	1375 reflections with *I* > 2σ(*I*)
plane graphite	*R*_int_ = 0.135
rotation method scans	θ_max_ = 25.0°, θ_min_ = 1.6°
Absorption correction: integration (*X-RED32*; Stoe & Cie, 2002)	*h* = −8→7
*T*_min_ = 0.797, *T*_max_ = 0.961	*k* = −15→15
12524 measured reflections	*l* = −29→29

### Refinement

**Table:**

Refinement on *F*^2^	Primary atom site location: structure-invariant direct methods
Least-squares matrix: full	Secondary atom site location: difference Fourier map
*R*[*F*^2^ > 2σ(*F*^2^)] = 0.069	Hydrogen site location: inferred from neighbouring sites
*wR*(*F*^2^) = 0.156	H-atom parameters constrained
*S* = 0.90	*w* = 1/[σ^2^(*F*_o_^2^) + (0.0426*P*)^2^] where *P* = (*F*_o_^2^ + 2*F*_c_^2^)/3
4206 reflections	(Δ/σ)_max_ < 0.001
298 parameters	Δρ_max_ = 0.14 e Å^−^^3^
0 restraints	Δρ_min_ = −0.16 e Å^−^^3^

### Special details

**Table:**

Geometry. All e.s.d.'s (except the e.s.d. in the dihedral angle between two l.s. planes) are estimated using the full covariance matrix. The cell e.s.d.'s are taken into account individually in the estimation of e.s.d.'s in distances, angles and torsion angles; correlations between e.s.d.'s in cell parameters are only used when they are defined by crystal symmetry. An approximate (isotropic) treatment of cell e.s.d.'s is used for estimating e.s.d.'s involving l.s. planes.
Refinement. Refinement of *F*^2^ against ALL reflections. The weighted *R*-factor *wR* and goodness of fit *S* are based on *F*^2^, conventional *R*-factors *R* are based on *F*, with *F* set to zero for negative *F*^2^. The threshold expression of *F*^2^ > σ(*F*^2^) is used only for calculating *R*-factors(gt) *etc*. and is not relevant to the choice of reflections for refinement. *R*-factors based on *F*^2^ are statistically about twice as large as those based on *F*, and *R*- factors based on ALL data will be even larger.

### Fractional atomic coordinates and isotropic or equivalent isotropic displacement parameters (Å^2^)

**Table:**

	*x*	*y*	*z*	*U*_iso_*/*U*_eq_	
Cl1	0.1715 (3)	0.89358 (11)	0.38214 (7)	0.1256 (8)	
S1	0.0165 (3)	0.51892 (13)	0.27709 (7)	0.1101 (7)	
N1	0.2484 (6)	0.4174 (3)	0.33380 (17)	0.0726 (14)	
N2	0.1837 (6)	0.5845 (3)	0.37084 (17)	0.0721 (14)	
N3	0.2157 (6)	0.5688 (3)	0.42568 (17)	0.0760 (15)	
O1	0.1257 (7)	0.7154 (3)	0.31249 (16)	0.1143 (18)	
C1	0.7867 (12)	0.1538 (6)	0.3653 (3)	0.127 (3)	
H1	0.8339	0.1724	0.3327	0.153*	
C2	0.8984 (11)	0.1442 (7)	0.4126 (4)	0.140 (3)	
H2	1.0198	0.1577	0.4106	0.168*	
C3	0.8345 (13)	0.1166 (6)	0.4591 (4)	0.110 (3)	
H3	0.9116	0.1100	0.4891	0.132*	
C4	0.6583 (13)	0.0977 (5)	0.4638 (3)	0.103 (2)	
H4	0.6128	0.0789	0.4966	0.123*	
C5	0.5461 (9)	0.1073 (4)	0.4178 (3)	0.091 (2)	
H5	0.4248	0.0943	0.4208	0.109*	
C6	0.6064 (11)	0.1348 (4)	0.3692 (3)	0.0798 (18)	
C8	0.4959 (9)	0.0466 (4)	0.2850 (2)	0.103 (2)	
H7A	0.6192	0.0327	0.2789	0.155*	
H7B	0.4325	0.0570	0.2513	0.155*	
H7C	0.4452	−0.0114	0.3034	0.155*	
C7	0.4812 (9)	0.1451 (4)	0.3194 (2)	0.0763 (18)	
C9	0.4947 (9)	0.2463 (4)	0.2867 (2)	0.0884 (19)	
H9A	0.5475	0.2368	0.2521	0.106*	
H9B	0.5516	0.3033	0.3064	0.106*	
C10	0.2863 (9)	0.1759 (4)	0.3310 (2)	0.0788 (18)	
H10A	0.2016	0.1192	0.3261	0.095*	
H10B	0.2737	0.2093	0.3656	0.095*	
C11	0.2862 (9)	0.2530 (4)	0.2835 (2)	0.0811 (18)	
H11	0.2394	0.2187	0.2508	0.097*	
C12	0.1991 (8)	0.3552 (4)	0.2899 (2)	0.0748 (18)	
C13	0.0734 (9)	0.3984 (4)	0.2568 (2)	0.097 (2)	
H13	0.0241	0.3657	0.2264	0.117*	
C14	0.1626 (8)	0.5056 (4)	0.3315 (2)	0.0730 (17)	
C15	0.1622 (8)	0.6881 (4)	0.3572 (2)	0.082 (2)	
C16	0.1878 (8)	0.7630 (4)	0.4043 (2)	0.0866 (19)	
H16A	0.3040	0.7515	0.4217	0.104*	
H16B	0.0976	0.7497	0.4303	0.104*	
C17	0.2259 (8)	0.4801 (4)	0.4471 (2)	0.0809 (19)	
H17	0.2139	0.4198	0.4265	0.097*	
C18	0.2573 (8)	0.4748 (4)	0.5056 (2)	0.0760 (17)	
C19	0.2719 (9)	0.5651 (5)	0.5343 (3)	0.108 (3)	
H19	0.2600	0.6288	0.5167	0.129*	
C20	0.3046 (11)	0.5631 (6)	0.5900 (3)	0.135 (3)	
H20	0.3144	0.6255	0.6089	0.162*	
C21	0.3222 (10)	0.4714 (7)	0.6168 (3)	0.123 (3)	
H21	0.3454	0.4713	0.6537	0.148*	
C22	0.3057 (8)	0.3774 (5)	0.5892 (2)	0.0807 (18)	
C23	0.2711 (8)	0.3776 (4)	0.5329 (2)	0.0745 (17)	
C24	0.2565 (8)	0.2805 (4)	0.5059 (2)	0.092 (2)	
H24	0.2392	0.2786	0.4687	0.111*	
C25	0.2679 (10)	0.1900 (5)	0.5343 (3)	0.106 (2)	
H25	0.2522	0.1270	0.5163	0.127*	
C26	0.3021 (10)	0.1887 (6)	0.5893 (3)	0.115 (3)	
H26	0.3126	0.1257	0.6076	0.138*	
C27	0.3200 (9)	0.2792 (7)	0.6158 (3)	0.106 (2)	
H27	0.3424	0.2779	0.6527	0.127*	

### Atomic displacement parameters (Å^2^)

**Table:**

	*U*^11^	*U*^22^	*U*^33^	*U*^12^	*U*^13^	*U*^23^
Cl1	0.187 (2)	0.0619 (9)	0.1246 (15)	−0.0055 (11)	−0.0343 (14)	0.0081 (10)
S1	0.1371 (17)	0.1007 (12)	0.0888 (12)	0.0283 (12)	−0.0443 (12)	−0.0148 (10)
N1	0.097 (4)	0.059 (3)	0.061 (3)	0.004 (3)	−0.011 (3)	−0.002 (2)
N2	0.106 (4)	0.058 (3)	0.052 (3)	0.011 (3)	−0.002 (3)	0.003 (2)
N3	0.107 (4)	0.069 (3)	0.051 (3)	−0.006 (3)	−0.009 (3)	0.004 (2)
O1	0.190 (5)	0.081 (3)	0.069 (3)	0.020 (3)	−0.018 (3)	0.014 (2)
C1	0.104 (7)	0.186 (8)	0.093 (6)	−0.001 (6)	0.000 (6)	−0.024 (5)
C2	0.092 (7)	0.232 (10)	0.097 (6)	−0.001 (6)	0.000 (6)	−0.026 (7)
C3	0.111 (8)	0.120 (6)	0.097 (6)	0.024 (6)	−0.013 (6)	−0.029 (5)
C4	0.123 (7)	0.086 (4)	0.097 (6)	−0.002 (5)	−0.018 (5)	0.005 (4)
C5	0.112 (6)	0.079 (4)	0.080 (5)	−0.007 (4)	−0.014 (5)	0.003 (4)
C6	0.096 (6)	0.067 (4)	0.077 (5)	0.006 (4)	0.001 (4)	−0.017 (4)
C8	0.129 (6)	0.091 (4)	0.089 (5)	0.017 (4)	−0.008 (4)	−0.029 (4)
C7	0.094 (6)	0.068 (4)	0.067 (4)	0.004 (3)	0.000 (4)	−0.011 (3)
C9	0.106 (6)	0.083 (4)	0.077 (4)	−0.005 (4)	0.010 (4)	−0.016 (4)
C10	0.099 (6)	0.071 (3)	0.067 (4)	−0.004 (4)	0.002 (4)	−0.003 (3)
C11	0.110 (6)	0.064 (4)	0.069 (4)	0.000 (4)	−0.005 (4)	−0.007 (3)
C12	0.105 (5)	0.059 (3)	0.060 (4)	−0.006 (3)	0.002 (4)	−0.006 (3)
C13	0.128 (6)	0.096 (4)	0.067 (4)	0.004 (4)	−0.023 (4)	−0.013 (4)
C14	0.108 (5)	0.063 (4)	0.046 (3)	−0.003 (4)	−0.013 (3)	0.003 (3)
C15	0.112 (6)	0.064 (4)	0.069 (4)	0.007 (3)	−0.008 (4)	0.008 (3)
C16	0.114 (6)	0.061 (3)	0.084 (4)	0.006 (3)	−0.010 (4)	0.008 (3)
C17	0.126 (6)	0.067 (4)	0.048 (4)	−0.013 (4)	−0.012 (3)	0.011 (3)
C18	0.100 (5)	0.067 (4)	0.060 (4)	−0.009 (3)	−0.005 (3)	−0.003 (3)
C19	0.166 (8)	0.077 (4)	0.077 (5)	−0.001 (4)	−0.025 (5)	−0.007 (4)
C20	0.198 (9)	0.118 (6)	0.087 (6)	−0.016 (6)	−0.029 (6)	−0.023 (5)
C21	0.163 (8)	0.145 (7)	0.061 (5)	−0.014 (6)	−0.021 (5)	−0.007 (5)
C22	0.089 (5)	0.107 (5)	0.046 (4)	0.000 (4)	−0.003 (3)	0.012 (4)
C23	0.082 (5)	0.079 (4)	0.063 (4)	−0.006 (3)	−0.002 (3)	0.006 (3)
C24	0.126 (6)	0.078 (4)	0.071 (4)	0.008 (4)	−0.010 (4)	0.012 (4)
C25	0.145 (7)	0.082 (5)	0.091 (5)	0.006 (4)	−0.003 (5)	0.021 (4)
C26	0.128 (7)	0.108 (6)	0.108 (7)	0.008 (5)	0.005 (5)	0.037 (5)
C27	0.107 (6)	0.138 (6)	0.073 (5)	0.010 (5)	−0.006 (4)	0.032 (5)

### Geometric parameters (Å, °)

**Table:**

Cl1—C16	1.766 (5)	C10—C11	1.541 (7)
S1—C13	1.686 (6)	C10—H10A	0.9700
S1—C14	1.720 (5)	C10—H10B	0.9700
N1—C14	1.301 (6)	C11—C12	1.476 (7)
N1—C12	1.392 (6)	C11—H11	0.9800
N2—C15	1.379 (6)	C12—C13	1.349 (7)
N2—N3	1.393 (5)	C13—H13	0.9300
N2—C14	1.412 (6)	C15—C16	1.523 (7)
N3—C17	1.257 (5)	C16—H16A	0.9700
O1—C15	1.190 (6)	C16—H16B	0.9700
C1—C6	1.380 (9)	C17—C18	1.470 (7)
C1—C2	1.425 (9)	C17—H17	0.9300
C1—H1	0.9300	C18—C19	1.364 (7)
C2—C3	1.321 (9)	C18—C23	1.422 (7)
C2—H2	0.9300	C19—C20	1.403 (8)
C3—C4	1.353 (9)	C19—H19	0.9300
C3—H3	0.9300	C20—C21	1.356 (8)
C4—C5	1.401 (8)	C20—H20	0.9300
C4—H4	0.9300	C21—C22	1.391 (8)
C5—C6	1.356 (8)	C21—H21	0.9300
C5—H5	0.9300	C22—C23	1.417 (7)
C6—C7	1.532 (8)	C22—C27	1.425 (8)
C8—C7	1.532 (6)	C23—C24	1.418 (7)
C8—H7A	0.9600	C24—C25	1.361 (7)
C8—H7B	0.9600	C24—H24	0.9300
C8—H7C	0.9600	C25—C26	1.384 (8)
C7—C9	1.539 (7)	C25—H25	0.9300
C7—C10	1.552 (7)	C26—C27	1.340 (8)
C9—C11	1.565 (8)	C26—H26	0.9300
C9—H9A	0.9700	C27—H27	0.9300
C9—H9B	0.9700		
			
C13—S1—C14	89.2 (3)	C9—C11—H11	110.0
C14—N1—C12	110.4 (4)	C13—C12—N1	113.8 (5)
C15—N2—N3	113.2 (4)	C13—C12—C11	126.9 (5)
C15—N2—C14	120.6 (4)	N1—C12—C11	119.3 (5)
N3—N2—C14	126.0 (4)	C12—C13—S1	111.8 (4)
C17—N3—N2	123.5 (4)	C12—C13—H13	124.1
C6—C1—C2	118.2 (8)	S1—C13—H13	124.1
C6—C1—H1	120.9	N1—C14—N2	123.4 (4)
C2—C1—H1	120.9	N1—C14—S1	114.8 (4)
C3—C2—C1	121.9 (8)	N2—C14—S1	121.8 (4)
C3—C2—H2	119.1	O1—C15—N2	122.4 (5)
C1—C2—H2	119.1	O1—C15—C16	123.6 (5)
C2—C3—C4	120.8 (8)	N2—C15—C16	114.1 (5)
C2—C3—H3	119.6	C15—C16—Cl1	110.6 (4)
C4—C3—H3	119.6	C15—C16—H16A	109.5
C3—C4—C5	118.2 (8)	Cl1—C16—H16A	109.5
C3—C4—H4	120.9	C15—C16—H16B	109.5
C5—C4—H4	120.9	Cl1—C16—H16B	109.5
C6—C5—C4	122.9 (7)	H16A—C16—H16B	108.1
C6—C5—H5	118.5	N3—C17—C18	117.9 (5)
C4—C5—H5	118.5	N3—C17—H17	121.0
C5—C6—C1	118.0 (6)	C18—C17—H17	121.0
C5—C6—C7	122.0 (7)	C19—C18—C23	119.4 (5)
C1—C6—C7	119.9 (7)	C19—C18—C17	119.1 (5)
C7—C8—H7A	109.5	C23—C18—C17	121.5 (5)
C7—C8—H7B	109.5	C18—C19—C20	120.8 (6)
H7A—C8—H7B	109.5	C18—C19—H19	119.6
C7—C8—H7C	109.5	C20—C19—H19	119.6
H7A—C8—H7C	109.5	C21—C20—C19	120.9 (6)
H7B—C8—H7C	109.5	C21—C20—H20	119.6
C6—C7—C8	109.1 (5)	C19—C20—H20	119.6
C6—C7—C9	116.8 (5)	C20—C21—C22	120.2 (6)
C8—C7—C9	113.0 (5)	C20—C21—H21	119.9
C6—C7—C10	115.1 (5)	C22—C21—H21	119.9
C8—C7—C10	113.7 (5)	C21—C22—C23	119.9 (6)
C9—C7—C10	88.0 (4)	C21—C22—C27	122.1 (6)
C7—C9—C11	89.2 (5)	C23—C22—C27	118.0 (6)
C7—C9—H9A	113.8	C22—C23—C24	118.5 (5)
C11—C9—H9A	113.8	C22—C23—C18	118.9 (5)
C7—C9—H9B	113.8	C24—C23—C18	122.7 (5)
C11—C9—H9B	113.8	C25—C24—C23	119.9 (5)
H9A—C9—H9B	111.0	C25—C24—H24	120.0
C11—C10—C7	89.7 (5)	C23—C24—H24	120.0
C11—C10—H10A	113.7	C24—C25—C26	122.2 (7)
C7—C10—H10A	113.7	C24—C25—H25	118.9
C11—C10—H10B	113.7	C26—C25—H25	118.9
C7—C10—H10B	113.7	C27—C26—C25	119.2 (7)
H10A—C10—H10B	110.9	C27—C26—H26	120.4
C12—C11—C10	118.3 (5)	C25—C26—H26	120.4
C12—C11—C9	119.2 (5)	C26—C27—C22	122.2 (6)
C10—C11—C9	87.4 (4)	C26—C27—H27	118.9
C12—C11—H11	110.0	C22—C27—H27	118.9
C10—C11—H11	110.0		
			
C15—N2—N3—C17	174.5 (6)	C15—N2—C14—N1	149.7 (6)
C14—N2—N3—C17	−1.6 (9)	N3—N2—C14—N1	−34.4 (9)
C6—C1—C2—C3	0.9 (12)	C15—N2—C14—S1	−31.5 (8)
C1—C2—C3—C4	−1.0 (13)	N3—N2—C14—S1	144.4 (5)
C2—C3—C4—C5	0.6 (12)	C13—S1—C14—N1	0.5 (5)
C3—C4—C5—C6	−0.2 (10)	C13—S1—C14—N2	−178.4 (5)
C4—C5—C6—C1	0.1 (10)	N3—N2—C15—O1	−176.4 (6)
C4—C5—C6—C7	−179.9 (5)	C14—N2—C15—O1	0.0 (10)
C2—C1—C6—C5	−0.4 (10)	N3—N2—C15—C16	3.1 (7)
C2—C1—C6—C7	179.5 (6)	C14—N2—C15—C16	179.4 (5)
C5—C6—C7—C8	−100.5 (7)	O1—C15—C16—Cl1	−4.2 (9)
C1—C6—C7—C8	79.6 (7)	N2—C15—C16—Cl1	176.3 (4)
C5—C6—C7—C9	129.9 (6)	N2—N3—C17—C18	−179.0 (5)
C1—C6—C7—C9	−50.1 (8)	N3—C17—C18—C19	0.8 (9)
C5—C6—C7—C10	28.7 (8)	N3—C17—C18—C23	−179.8 (6)
C1—C6—C7—C10	−151.2 (6)	C23—C18—C19—C20	1.6 (10)
C6—C7—C9—C11	−135.0 (6)	C17—C18—C19—C20	−179.0 (7)
C8—C7—C9—C11	97.2 (5)	C18—C19—C20—C21	−0.1 (13)
C10—C7—C9—C11	−17.8 (4)	C19—C20—C21—C22	−0.8 (13)
C6—C7—C10—C11	136.9 (5)	C20—C21—C22—C23	0.2 (11)
C8—C7—C10—C11	−96.2 (5)	C20—C21—C22—C27	−178.8 (8)
C9—C7—C10—C11	18.1 (4)	C21—C22—C23—C24	179.5 (6)
C7—C10—C11—C12	−139.8 (5)	C27—C22—C23—C24	−1.4 (9)
C7—C10—C11—C9	−17.8 (4)	C21—C22—C23—C18	1.2 (9)
C7—C9—C11—C12	139.1 (5)	C27—C22—C23—C18	−179.7 (6)
C7—C9—C11—C10	17.9 (4)	C19—C18—C23—C22	−2.1 (9)
C14—N1—C12—C13	−1.6 (7)	C17—C18—C23—C22	178.5 (6)
C14—N1—C12—C11	177.6 (5)	C19—C18—C23—C24	179.7 (6)
C10—C11—C12—C13	−127.6 (7)	C17—C18—C23—C24	0.3 (9)
C9—C11—C12—C13	128.4 (6)	C22—C23—C24—C25	2.9 (9)
C10—C11—C12—N1	53.3 (8)	C18—C23—C24—C25	−178.9 (6)
C9—C11—C12—N1	−50.8 (7)	C23—C24—C25—C26	−3.2 (11)
N1—C12—C13—S1	2.0 (7)	C24—C25—C26—C27	1.8 (12)
C11—C12—C13—S1	−177.2 (5)	C25—C26—C27—C22	−0.3 (12)
C14—S1—C13—C12	−1.4 (5)	C21—C22—C27—C26	179.2 (8)
C12—N1—C14—N2	179.4 (5)	C23—C22—C27—C26	0.2 (10)
C12—N1—C14—S1	0.5 (6)		

### Hydrogen-bond geometry (Å, °)

**Table:**

Cg1 and Cg2 are the centroids of the C12/C13/S1/C14/N1 and C22–C27 rings, respectively.

**Table:**

*D*—H···*A*	*D*—H	H···*A*	*D*···*A*	*D*—H···*A*
C13—H13···O1^i^	0.93	2.41	3.243 (7)	148.
C8—H7A···Cg1^ii^	0.96	2.94	3.748 (3)	143
C16—H16B···Cg2^iii^	0.97	2.93	3.740 (8)	142

Symmetry codes: (i) −*x*, *y*−1/2, −*z*+1/2; (ii) −*x*+1, *y*−1/2, −*z*+1/2; (iii) −*x*, −*y*+1, −*z*+1.
